# Overview on brain function enhancement of Internet addicts through exercise intervention: Based on reward-execution-decision cycle

**DOI:** 10.3389/fpsyt.2023.1094583

**Published:** 2023-02-02

**Authors:** Hao Chen, Guijun Dong, Kefeng Li

**Affiliations:** ^1^Department of Sports, Quzhou University, Quzhou, China; ^2^Department of Graduate School of Education, Shandong Sport University, Jinan, China; ^3^Department of Medicine, Quzhou College of Technology, Quzhou, China

**Keywords:** exercise, Internet addiction, brain function, reward system, executive system, decision-making system

## Abstract

Internet addiction (IA) has become an impulse control disorder included in the category of psychiatric disorders. The IA trend significantly increased after the outbreak of the new crown epidemic. IA damages some brain functions in humans. Emerging evidence suggests that exercise exerts beneficial effects on the brain function and cognitive level damaged by IA. This work reviews the neurobiological mechanisms of IA and describes the brain function impairment by IA from three systems: reward, execution, and decision-making. Furthermore, we sort out the research related to exercise intervention on IA and its effect on improving brain function. The internal and external factors that produce IA must be considered when summarizing movement interventions from a behavioral perspective. We can design exercise prescriptions based on exercise interests and achieve the goal of quitting IA. This work explores the possible mechanisms of exercise to improve IA through systematic analysis. Furthermore, this work provides research directions for the future targeted design of exercise prescriptions.

## 1. Introduction

The concept of Internet addiction (IA) was first introduced by Ivan Goldbery, an American psychiatric researcher. Later, psychologist Kimberly Young first defined IA as a “behavior-control disorder” that did not involve intoxication (1). With the study of the definition of IA, it gradually extended from psychology to the field of psychiatry. However, no uniform definition of this concept has been established to date. The academic community still remains in disagreement with this terminology, and several terms are obscured. Existing research commonly defines IA as compulsive behaviors and cognitions associated with Internet use that result in significant distress in daily life (2). With an Internet penetration rate of over 94.9% in China, the prevalence of IA is on a surge (3). In particular, the proportion of underage Internet users with Internet dependency psychology has been as high as 17.3% (4). This condition can lead to social withdrawal tendencies (5), suicidal tendencies (6), and a range of other behavioral and psychological problems in minors. At present, the brain reward circuit function, decision-making ability, and executive ability of IA patients are significantly lower than those of healthy individuals (7–10).

Although clinical methods for treating IA are available, such as pharmacotherapy and psychotherapy, they are still deficient to a certain degree. Studies have shown that medication reduces the craving for the Internet by suppressing the activity in some areas of the brain of people with IA. This mechanism brings the patient’s brain activity into a rewarding balance. Then, the patient’s brain activity reaches a rewarding balance. Although this method can improve IA, its exogenous nature leads to severe side effects and high dependence on the patient. Psychotherapy consists in improving the cognition of IA patients through psychological counseling, which leads to abstinence from IA. However, this method suffers from the disadvantages of long periodicity and low abstinence (11). Exercise is gradually becoming a major means of intervention for IA due to its unique advantages of high withdrawal and few side effects. In comparison with pharmacological and psychological interventions, the sensations generated during the exercise intervention can replace much of the experience of internet use, thus allowing patients to return to real life more quickly. Medication is frequently used with fluoxetine and bupropion to eliminate anxiety in patients with IA. However, this effect is not entirely effective because most anti-anxiety approaches require active neurogenesis (12). Patients with IA are frequently accompanied by a sense of stigma, and medication makes them psychologically resistant. Exercise not only helps active neurogenesis but also greatly reduces the patient’s sense of stigma. In a meta-analysis, exercise interventions were more effective than medication (13). A 1 year exercise intervention on IA patients was found to effectively improve IA and its negative effects on body and mind, and it significantly improves the patient’s interpersonal relationship, mental health, and IA symptoms and reduces the risk of IA (14). Although exercise can be effective in improving IA, understanding of the underlying neural mechanisms of exercise to alleviate IA is scarce. Current research has found that exercise may alleviate IA by improving neuro molecular mechanisms, including increasing the activation of the brain-derived neurotrophic factor (BDNF) (13), 5-hydroxytryptamine (5-HT), and noradrenaline (14). These neural molecules can improve the structure and function of synapses between neurons and influence the development of new neurons. In turn, this situation can compensate and restore the damage caused by IA to brain function (15). With the outbreak of COVID-19, the frequency of internet use among adolescents has dramatically increased, in contrast with a decrease in social and outdoor exercise, leading to an increase in the number of IA patients (16). We must explore the mechanisms of the effectiveness of exercise interventions in IA. The databases searched included PubMed, Google Scholar, Sport Discus, Web of Science, Scopus, and China National Knowledge Infrastructure. The keywords used in the search include the following: internet addition, internet dependency, internet addition, internet dependency, pathological internet use, on-line dependency, problematic internet use, excessive internet use, exercise, physical fitness, movement, physical activity, sports, neurogenesis, reward system, execution system, decision-making system, brain, brain function, and cognition. From a neurobiological perspective, we analyze the mechanisms by which exercise improves brain function in patients with IA. This overview also summarizes the current state of research on exercise interventions for IA. This work also suggests the possible neurobiological mechanisms for an exercise intervention in IA rehabilitation, providing directions for future exploration of more effective IA treatments and means of prevention.

## 2. Formation of IA

We need to grasp the process of IA production to effectively explore the withdrawal mechanisms of exercise interventions for IA. Young (17) proposed the ACE (Availability, Controllability, Excitability) model to illustrate the formation process of IA in terms of three factors: availability, controllability, and excitability (Table 1). Availability refers to the accessibility of the Internet, the attractiveness of the Internet content, and the inner satisfaction of using the Internet. Controllability refers to the degree of control over the use of the Internet, a stage of withdrawal behavior that creates inner restlessness. Excitability refers to the dependence on the Internet and the difficulty in suppressing the strong urge to go online (17).

**TABLE 1:** Formation process of Internet addiction (IA) in the Availability, Controllability, Excitability (ACE) model (Kimberly S. Young).

Stage	Features	Performance
Indulgence and revelry	NAcc is unusually active, and the reward system is shifting from natural rewards to behavioral inducements.	Hard to resist the urge to use the Internet.
Indulgence and revelry	The dopaminergic system causes an imbalance in the reward system and a shift in motivation to use the Internet from pleasure seeking to alleviating negative emotions.	Severe weakening of inhibitory control and withdrawal syndrome.
Desire and seek	Increasing imbalance in the reward system and a dramatic increase in the thirst for the Internet.	Unstoppable urge to surf the Internet, which seriously affects daily life and impairs the brain function.

First, neurons of multiple transmitters, such as dopamine (DA) in the ventral tegmental area (VTA), project to the nucleus accumbens (NAcc) *via* efferent nerve fibers during the embryonic phase of IA formation. However, the excitability of neurons within the NAcc is governed by G protein signaling within the prefrontal cortex (PFC), which causes an increased release of DA in the NAcc (18). After repeated stimulation, NAcc showed abnormal activity (19). This condition causes the reward system in people’s brains to be activated. At the molecular level, this adaptation to the network environment is a reflection of plasticity changes. This change mainly affects the way the neurotransmission of DA and glutamate is integrated to enhance or diminish the inter-synaptic communication (20). Accordingly, individuals can gain full satisfaction in using the Internet. This change also upsets the balance of natural rewards and promotes the formation of IA.

Second, as people unusually use the Internet for longer periods of time, the rewards system becomes more sensitive to the use of the Internet. This situation occurs because ΔFosB (a marker of repetitive neuronal activity associated with reward) continues to accumulate when IA patients use the Internet. After removal of the stimulus, this marker remains in the brain for a long time (21). This phenomenon not only enhances the motivation and sensitivity of IA patients to rewards (22) but also reduces the aversion sensitivity (22). When patients at this stage do not use the internet, they become upset by withdrawal. However, the rewards obtained from occasional Internet use no longer maintain the homeostasis of DA in the brain. Patients pursue longer use of the Internet to satisfy pleasure or to reduce negative emotions. This behavior enabled the patients to receive more dopamine reward, causing them to normalize their dopaminergic energy. Nonetheless, this repeated stimulation can contribute to the development and maintenance of IA (23), creating a vicious cycle that can lead to an imbalance in the reward system.

**FIGURE 1:**
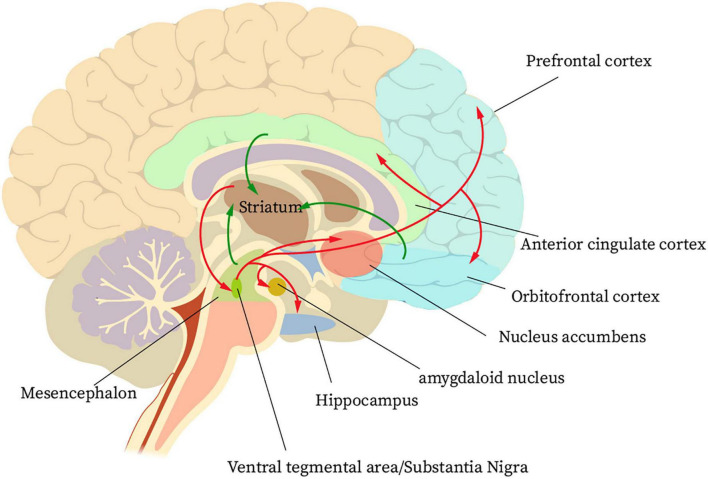
The basic neural circuit of reward for internet addicts.

Finally, when the patient has formed a habit of using the Internet, the end stage of IA formation has been reached at this time. During this phase, glutamatergic projections from the anterior cingulate gyrus and orbitofrontal lobe to the NAcc cause adaptive changes in nerve cells. However, this change reduces the value of natural rewards in the patient’s brain and instead enhances the pursuit of addictive behaviors (18). A reward circuit is formed in the striatum cortex regarding IA (Figure 1). First, large amounts of dopaminergic input from the orbitofrontal cortex, the anterior cingulate cortex, and the midbrain pass through the nerve fibers to the striatum. The striatum then projects information to the ventral pallidum and the VTA/substantia nigra. Finally, the ventral pallidum and the VTA/substantia nigra projects information to the PFC, NAcc, hippocampus, and amygdala. Accordingly, a reward cycle is created (24). The reward system for IA patients will continue to be out of balance during this phase. This condition is due to a dramatic decrease in dopamine D2 receptor and dopamine transcript levels in the patient’s NAcc (25). Such a circumstance, coupled with changes in neuroplasticity mediated by glutamatergic signaling, further disrupts the regulation of reward and emotion circuits in the PFC region (26). At this time, if the Internet is not used for a long time, the IA patients will have an uncontrollable urge to go online, which will seriously affect their normal life.

## 3. Abnormalities in brain regions associated with reward, executive, and decision systems in IA patients

Currently, researchers commonly use different neurovisualization methods, such as electroencephalography (EEG), positron emission computed tomography (PET), single photon emission computed tomography (SPECT), functional magnetic resonance imaging (fMRI), and structural magnetic resonance imaging (sMRI), to study IA. The structure and function of the IA patients’ brains are extensively studied by these methods when they are in task conditions or resting states. Based on the assessment of the gray matter volume and functional connectivity, the researchers found that the IA patients had some degree of impairment in three systems: reward, executive, and decision-making. This alteration in the brain structure and functional connectivity of the various regions results in abnormal brain function in IA patients.

### 3.1. Reward system

The reward system is thought to be the most critical neurogenetic basis for addiction. The key structures of this system are the anterior cingulate cortex, orbitofrontal cortex, dorsal prefrontal cortex, limbic striatum, VTA, thalamus, and midbrain (27). Neuroimaging studies have shown a significant decrease in gray matter volume and changes in white matter fiber tracts in the PFC region of IA patients (28). The functional and structural connectivity of the pathway between VTA and NAcc to the medial orbitofrontal cortex was significantly reduced. However, this abnormal performance affects sensory integration and sensory information processing (29). Dopamine neurons within the VTA project to the NAcc and PFC as part of the limbic pathway in the midbrain and are critical for reward. The diminished function of the reward system in IA patients was demonstrated by fMRI (30, 31). This result is consistent with the decreased availability of the DA receptors in the striatum of IA patients observed by PET (32). In addition, patients with IA have reduced levels of dopamine D2 receptor availability within various subregions of the striatum (33). This reduction must be rewarded by more network participation to normalize its own dopaminergic. At this time, the patient develops a higher craving for the Internet, which enhances activation of the left superior frontal gyrus of the brain (34). This condition It can make IA patients more sensitive to getting rewards and ignore the adverse effects. IA patients will prefer immediate rewards to delayed but more favorable rewards (35, 36). Immediate rewards meet the physical and psychological needs of the patient. Upon receiving an immediate reward, dopamine neurotransmitters are released in the brains of IA patients, giving them a sense of psychological and physical satisfaction. Thus, we can restore the brain’s reward system by improving the brain’s dopaminergic levels, which improves IA.

### 3.2. Execution system

The executive function of the brain plays an important role when people are faced with a multitude of problems and need to quickly select the necessary information. This function enables many cognitive processing processes to simultaneously operate to optimize brain cognition to make optimal choices. The study found a reduction in the volume of gray matter in some brain regions in IA patients, including the dorsolateral prefrontal cortex, orbitofrontal cortex, and cingulate gyrus (37). These regions are thought to be important brain areas involved in cognitive and executive control (38–40). This notion suggests that the executive dysfunction in IA patients may be related to abnormalities in their brain structure. However, changes in the brain structure lead to a decrease in nerve cell activity in the brain regions, which results in impaired brain function. In the Stroop task, impaired executive ability mechanisms were revealed by recording relevant brain potentials in IA patients. This injury does not successfully inhibit brain activation (41). However, the continued activation of the brain may cause IA patients to lose the ability to judge short-term benefits versus long-term damage, driving them to seek immediate rewards (42). Addiction is not only associated with the breakdown of the structure and function of separate brain regions but also has a relationship with the functional connectivity of individual brain regions. The functional connectivity of the executive control network was found to be significantly lower in IA patients than in healthy individuals by fMRI scans (43). A reduced anti-correlation was observed between the medial prefrontal cortex and the anterior cingulate gyrus. These reductions in functional connectivity may cause dysregulation of cognitive control networks and reduce cognitive efficiency (30). Another study found impaired inhibitory neuromodulation of PFC by putamen when the IA patients performed a cue craving task (42). Furthermore, the study of IA was refined. The dorsolateral prefrontal cortex, cingulate gyrus, and medial prefrontal cortex brain regions are more active in IA patients when they are in the Internet state. This activation results in diminished cognitive executive control and a strong craving response to the network. Patients with IA exhibit longer reaction times and higher error rates in task states compared with normal individuals (41). Moreover, the Internet thirst is higher in the quiet state. This cognitive impairment increases the likelihood that IA will be difficult to control (44).

### 3.3. Decision-making systems

Decision-making is the ability of an individual to make the best choice from among multiple options. The decision-making process is complex and characterized by risk aversion. Addicts reap high rewards and often ignoring negative consequences. This tendency occurs because of abnormalities in decision-making and impulse control (45, 46). Abnormal decision-making behaviors may reflect dysfunctions in the structure and function of the brain’s decision-making system. Studies have shown that patients with IA have reduced white matter density and gray matter density in the inferior frontal gyrus and insula (9). The inferior frontal gyrus is an important brain region for assessing the relationship between reward and risk (47). Meanwhile, insula integrates mutual perception states into conscious sensing and involves risk and reward in the decision-making process (48). Damage to the inferior frontal gyrus and insula inevitably produces unfavorable decision-making. Structural changes in the associated brain regions can also cause abnormalities in brain function. This abnormality is manifested by less oxygen level-dependent signal activation in the inferior frontal gyrus (49) and enhanced functional connectivity in the insula. However, the functional connectivity of the insula is positively correlated with impulsivity (50). Consequently, patients with IA show poorer control in the face of greater attraction. In addition, the IA symptoms are accompanied by a range of psychological problems, such as depression, social fear, and suicidal tendencies. These problems may also be related to dysfunction in the conversion of somatic and emotional sensory signals (51), which affects cognitive abilities. The likelihood of poor decision-making is increased in IA patients. Another study found that IA patients performed significantly lower in terms of decision quality by using physiological markers of late positive potential (LPP) and identifying the source of LPP (35). In summary, the IA can seriously affect decision-making and risk assessment capabilities. Patients with IA exhibit a reduced aversion to risk and are unable to adequately assess the potential loss behind risky choices. The patients are also unable to effectively control their reward-seeking behavior, which leads to higher risky choices (49). This situation may be one of the reasons why IA patients continue to use the Internet. The damage to brain areas caused by IA differs from substance addiction because it tends to be more of a spontaneous processing of information by the brain (36). Specifically, the decision-making and risk-assessment abilities of IA patients can be enhanced by human intervention.

In summary, patients with IA exhibit higher impulsivity, reduced ability to delay gratification and assess risk, altered expected outcomes for risky situations and decisions, and the presence of risk-taking tendencies. This performance behavior is closely related to abnormalities in the brain structure and function. Although the damage to the brain from IA is described in this work in three systems, evidence shows that they are functionally related and could potentially interact to cause inappropriate or maladaptive behavior (52). The process is intertwined from the generation of rewarding affective and cognitive responses to the reduction of executive function and inhibitory control and to the generation of adverse decisions. Given that the IA patients respond to cues and crave networks, the dysfunctional interaction between their brain’s executive function impairment and contextual reward seeking may promote adverse decision-making. Despite knowing the negative consequences of long-term Internet use, people with IA seek short-term satisfaction to reduce cravings and increase emotional decision-making through Internet use. People will still use the Internet for long periods of time.

## 4. Exercise intervention to improve IA

Internet addiction is a behavioral addiction that triggers structural changes in the brain’s reward system, giving the brain transcendental pleasure. However, this condition can also cause damage to the structure and function of the brain’s reward, executive, and decision-making systems. Changes in the patient’s reward circuitry are a key factor in addiction and a pathway to recovery. However, no standard of care is available for the treatment of IA. Currently, the four commonly used treatments are exercise prescription intervention, group counseling, general psychological intervention, and cognitive behavioral therapy. Exercise prescription interventions are currently the optimal solution for interventions in IA (53). Exercise prescription interventions are easier and more economical than the other three interventions. The physical sensations induced by prolonged exercise not only replace the satisfaction generated by Internet use but also improve the brain function (54), causing withdrawal from IA.

Some studies have shown that regular participation in physical activity is not only effective in relieving IA but also in preventing its occurrence. We should intervene by controlling the duration, intensity, and frequency of exercise. In comparison with acute exercise, the duration of chronic exercise has a moderating role in addiction symptomatology, and chronic exercise for at least 12 weeks is most beneficial in reducing addictive behaviors. Improving reward and inhibitory control structures and functions in the associated brain through exercise requires longer exercise programs (e.g., 12+ weeks) for brain-related morphological adaptations to occur (55). This study determined the minimum period of intervention in which exercise could effectively interfere with IA. Aerobic intermittent exercise contributes to recovery of the PFC function and withdrawal from addiction (56), and moderate intensity exercise reduces craving levels (57). However, the experimental form of this exercise intervention is rather boring and homogeneous, and the long-term intervention cannot create a strong desire for exercise in IA patients, and it cannot replace the satisfaction brought by the network. To slow down or quit IA, people with IA can acquire exercise skills, form exercise habits, develop an interest in exercise, and reduce cyber behavior. A study has proposed the concept of exercise addiction replacement IA. Forty IA high school students were developed to master skills, exercise interest, and form exercise habits through a year-long exercise intervention (the exercise was led by IA patients). After the intervention experiment, the researchers conducted a 9 months follow-up. They found that patients with mild IA showed significant improvement after the intervention, and patients with severe IA also showed significant improvement after 3 months of intervention. A total of 45 IA adolescents had formed exercise habits and quit IA. It demonstrated the effectiveness, persistence, and low recurrence rate of exercise interventions for IA and the feasibility of exercise addiction replacement for IA (14). However, there are differences in the causes of IA, and different living environments, personalities, and other factors play a role in the development of IA to different degrees. Accordingly, exercise interventions should be appropriately combined with psychological interventions and targeted exercise choices to more effectively improve IA. In conclusion, physical exercise can effectively strengthen the muscle strength of IA patients, enhance self-confidence, develop tough willpower, enhance interpersonal skills in real life, and reduce the impulse to go online. Therefore, in future exercise interventions, we need to design exercise prescriptions according to their exercise interests and in response to the internal and external factors that produce IA. Such work will be more precise and effective to achieve the goal of withdrawal from IA.

## 5. Mechanisms of exercise enhanced neuroplasticity

Neuroplasticity refers to the ability of the nervous system to produce adaptive responses by improving its structure, function, and connectivity following stimulation (58). This feature involves a variety of mechanisms, such as neurogenesis, synaptic plasticity, and dendritic plasticity (59). The developing brain is more neuroplastic than the adult (60). Some studies have shown that neuroplasticity can be induced through physical exercise after an adult brain injury (61, 62).

### 5.1. Neurogenesis

Neurogenesis is one of the main manifestations of brain plasticity at the cellular level. It refers to the generation of new neurons by neural stem cells or neural progenitor cells under induction (63). Neurogenesis still exists in the adult central nervous system, and the hippocampal dentate gyrus maintains the ability to generate new neurons throughout life (64). Aerobic exercise enhances the proliferation, migration, survival, and differentiation of neural stem cells or neural progenitor cells in the dentate gyrus portion of the hippocampus, promoting neurogenesis (65). In synaptic transmission, multiple presynaptic axons may innervate a postsynaptic cell. Aerobic exercise disrupts the balance of competition between synapses, causing changes in synaptic circuits and a reorganization of synaptic connections, which affects the structure and function of the brain (66). Furthermore, aerobic exercise alters the hippocampal gray matter volume, possibly by changing neurogenesis, glial cell production, or the strength of interneuronal connections (synaptogenesis), thereby modifying existing cortical structures (67).

### 5.2. Synaptic plasticity

Synaptic plasticity includes short-term synaptic plasticity and long-term synaptic plasticity. Short-term synaptic plasticity consists mainly of facilitation, depression, and potentiation. Meanwhile, long-term synaptic plasticity is mainly manifested in the form of long-term potentiation (LTP) and long-term depression (68). Exercise increases the synaptic strength of the perforant pathway connecting the entorhinal cortex to the dentate gyrus, which can enhance LTP (69). In neurodegenerative diseases, cognitive decline is accompanied by a reduction in hippocampal neurogenesis (70). The main pathological features are neurofibrillary tangles and the formation of amyloid-beta (Aβ) plaques (71). Aβ can inhibit LTP in the dentate gyrus *via* N-methyl-D-aspartate receptor (NMDA) signaling (72). At the post-synaptic regulation level, the BDNF-tyrosine kinase receptor B signal induces NMDA phosphorylation and increases the opening of ion channels. The influx of calcium ions can activate Ca2+/calmodulin-dependent protein kinase II (CaMKII) to maintain enhanced synaptic efficiency (73). In a rat model, exercise protects synaptic transmission and LTP in the dentate gyrus and normalizes the levels of synaptic plasticity-related molecules, such as CaMKII, calcineurin, and BDNF (74). Thus, exercise enhances synaptic plasticity. In addition, in a rat model, exercise was found to increase the levels of synaptophysin, postsynaptic density-95, microtubule-associated protein, and tau protein in the hippocampus (75). An increase in these indicators may facilitate the repair and reconstruction of nerve damage and remodel brain function.

### 5.3. Dendritic plasticity

Dendritic morphology is a key factor in the proper functioning of the nervous system, which shows great diversity in different types of neurons (76, 77). The complexity of this factor directly affects the function of the nerve tissue. Dendritic remodeling can be observed under physiological conditions and following neuropathological stimulation, suggesting that dendrites are dynamic structures (77). Degeneration of dendrites and loss of synapses may be one of the mechanisms of brain damage in neurological disorders, such as Parkinson’s, Alzheimer’s, depression, and other brain injuries (78–80). In a mouse model with Parkinson’s, the density of dopamine receptor-containing dendritic spines is reduced. However, exercise prevented the loss of density of dendritic spines of spiny neurons in the striatum and upregulated the expression of postsynaptic density-95 protein, resulting in improved symptoms in Parkinson’s mice (81). Evidence shows that stress-induced reductions in neurogenesis, dendritic atrophy, and loss of spines in hippocampal and prefrontal cortical neurons are involved in the pathophysiology of depression (79). The use of an animal model of corticosterone-induced stress demonstrates that physical exercise can reverse inhibited hippocampal neurogenesis and reduce spinal density. Running the wheel enhanced the hippocampal neurogenesis, increased the dendritic length, and restored the spinal density, thereby improving the depressive activity in a rat stress model (82). Recent studies have found that exercise can reverse prefrontal cortex abnormalities in dendritic length and branching (83). These exercise-induced benefits suggest that exercise can be used as a treatment modality for some neurological disorders to improve the condition of patients.

## 6. Exercise improves brain damage in Internet addicts

We believe that exercise is the most effective way to promote physical health. The neurotransmitters and neurotrophic factors that benefit the brain are synthesized after physical exertion (84, 85). These factors may delay the progression of neurodegenerative diseases and mental disorders (86) and promote a sense of pleasure and wellbeing, thereby promoting physical and mental health. Studies have shown that exercise-induced neurotransmitter release and elevated neurotrophic factor activity contribute to neuroplasticity (87) and normal cortical activity (88). Glial cell-derived neurotrophic factor (GDNF), 5-HT, and vascular endothelial growth factor production are important not only for neurogenesis but also for neuronal maintenance and prevention of psychological disorders (89). These neurotrophic factors are induced by muscle contraction, causing changes that promote the formation of a more plastic and adaptive brain and enable the maintenance of the brain structure and function.

### 6.1. Changes in neurotransmitters and their receptors in the brain

#### 6.1.1. Dopamine and its receptors

Dopamine and its D2 receptors are closely associated with activation levels in the brain regions, such as the PFC, anterior cingulate gyrus, and insula, which are responsible for the reward, execution, and decision-making. However, dysregulation of the dopamine system underlies IA behavior and craving, ultimately causing uncontrolled behavior in IA patients. Studies have shown that exercise alters circuits of dopamine in the midbrain-striatum and those involved in emotional assessment (90). Exercise increases the synthesis and release of DA and stimulates neuroplasticity. These receptors can act as powerful triggers not only for peripheral adaptation processes (e.g., cardiovascular and musculoskeletal adaptation) but also for brain plasticity (91). People’s chronic addiction to the Internet can cause blockage of dopamine release pathways in the brain. After a motor intervention, the IA reward circuit triggered by DA was not activated, and the release of DA and the utilization of its receptors were significantly decreased (92). In this case, IA patients’ satisfaction was suppressed, thereby reducing their participation in the network. Moreover, active aerobic exercise not only enhances dopamine conversion but also increases its density, inducing neuroadaptation and improving control. The adherence of IA sufferers to long-term voluntary exercise can promote the release of DA in the striatum, improve DA transmission, and increase dopamine D2 receptor activity. Furthermore, such adherence can improve the dopaminergic system of IA patients, maintain midbrain dopaminergic homeostasis, and normalize the reward system (93). In addition, an association exists between low levels of monoamine neurotransmitters and negative mood in patients with IA. Long-term aerobic exercise increases the release of monoamine neurotransmitters (dopamine and norepinephrine in the hypothalamus), thereby reducing feelings of stress and anxiety (94) and cyber behavior.

#### 6.1.2. Glial cell derived neurotrophic factor

Glial cell-derived neurotrophic factor is a neurotrophic factor that is essential in maintaining the development, survival, and maintenance of dopaminergic neurons in several brain regions and midbrain dopaminergic neurons (95). Although the role of GDNF in IA is unknown, studies on IA demonstrated that GDNF is negatively correlated with the degree of IA and is expected to be a target for the IA treatment (96). Studies have shown that GDNF promotes the survival and differentiation of midbrain dopaminergic neurons, which may be related to midbrain tyrosine hydroxylase activity. These changes may alter the synapses and responsiveness of the limbic dopaminergic system in the midbrain of IA patients, ultimately weakening the stimulus or reward pathways and neural adaptations associated with addiction (97). GDNF is also important in the development and maintenance of spinal motor neurons and midbrain dopaminergic neurons (98). Exercise can affect the function and structure of parts of the brain by modulating the levels of trophic factors, such as GDNF (99), to regenerate damaged axons and alleviate symptoms in IA patients. In research regarding the mode of movement, the GDNF expression is regulated in an activity-dependent manner, and the expression may depend on the recruitment of muscle fibers. After a low-intensity exercise, the increased expression of GDNF was observed in the soleus (predominantly slow contraction). By contrast, this expression is reduced in the extensor longus digitorum (predominantly fast contracting) (100). We should increase the recruitment of fast muscle fibers to obtain higher GDNF levels and achieve the effect of treating IA.

#### 6.1.3. 5-Hydroxytryptamine and its receptors

5-hydroxytryptamine, also known as serotonin, is an important inhibitory neurotransmitter in the brain. In neural circuits, 5-HT neurons in the dorsal raphe nucleus (DRN) can receive projections not only from the forebrain and limbic system but also to these brain regions, forming complex neural loops (101–103). In the reward neural circuit, the 5-HT neurons within the DRN form the reward neural circuit by interconnecting with dopamine neurons within the VTA, glutamatergic neurons within the PFC, and gamma-aminobutyric acid neurons within the NAcc. In the exploration of addictive disorders, a decrease in the activity of the 5-HT system was found to be associated with it. 5-HT2A receptors were found to be reduced in the left and right temporal cortices of IA patients by PET (32). The decreased levels of this receptor can increase the anxiety in addicted individuals and may trigger compulsive online behavior. In mice experiments, exercise may promote 5-HT2A receptor mediated γ-aminobutyric acidergic inhibition of input to the amygdala, resulting in anxiolytic effects on psychological stress (104). In addition, acute aerobic exercise can enhance individual response inhibition by increasing the expression of 5-HT, thereby suppressing the urge to go online in IA patients (105). This condition indicates that the expression of 5-HT and its receptors can be increased by acute exercise, thereby reducing the anxiety associated with not being online. Furthermore, the improvement of inhibitory function in IA patients is promoted, and the strong urge to go online is suppressed.

### 6.2. Structure and function of the brain regions associated with reward, executive, and decision-making systems

#### 6.2.1. Reward system

Prolonged, repeated voluntary exercise produces a reward state that persists after exercise has ceased and induces plastic changes in the limbic reward pathways of the midbrain (106). This change is the basis for the formation and consolidation of addictive behaviors. After exercise, the GDNF levels in the striatum improve (107), and ΔFosB (108) and delta-opioid receptor (DOR) expression is enhanced. ΔFosB, which is a molecule involved in addiction to repeated stimuli, is an important transcription factor. The activation of this factor after exercise induces habituation in IA patients and causes changes in the transcription of genes in their reward circuit. Activation of DOR promotes the release of DA in NAcc (109) and the ability to synthesize DA. This condition may help in enhancing the dopamine neurotransmission in NAcc (106) because the striatal adenosine and dopaminergic systems are targets of exercise-generated neuroplasticity. Meanwhile, adenosine is the primary regulator of striatal activity. Exercise can affect the striatal and dopaminergic systems by influencing the activity of adenosine and DA. A moderate increase in mRNA for dopamine in striatal subregions was found after exercise *via* radioactive or double-labeled fluorescence *in situ* hybridization assays. By contrast, the mRNA levels of adenosine receptors and receptors in the striatum were reduced (110). Clinical studies suggested that the reduction in mRNA levels of adenosine receptors after exercise may be associated with a reduction in addictive properties. The link between exercise to improve IA and striatal adenosine receptor mRNA needs to be further explored. Additional studies have shown that the interaction between BDNF and dopamine signaling in the limbic circuits of the midbrain is critical in rewarding behavior (111, 112). After exercise, an increased availability of dopamine D2 receptors was observed in the posterior shell nucleus (113), and the upregulation of BDNF molecules stimulated the neuronal growth, thus effectively improving the reward system. After habitual exercise, the neuroplasticity in the reward pathway may contribute to the continuation of exercise, which is associated with the neurotransmission of DA. This neurological enhancement would inevitably improve the brain structure and function, especially an increase in PFC volume associated with reward (114). Such a condition may be effective in improving the brain function in IA patients. This fact is evidenced by a reduction in the incidence and severity of IA and by promoting successful withdrawal from IA.

#### 6.2.2. Execution system

Patients with IA have severely impaired the executive function, resulting in an unquenchable desire for cyber behavior (43). By contrast, exercise triggers or enhances complex neurobiological processes to improve the executive function in the brain. Current researchers can observe changes in the hippocampus, anterior cingulate cortex, and dorsolateral prefrontal cortex structures accompanied by alternations in the cognitive and executive functions by sMRI. After exercise, the subjects demonstrated an increase in the hippocampal volume, and a larger hippocampal volume mediated the association between adaptation and spatial working memory (115). The volume of the subjects’ anterior cingulate gyrus also increases with movement, thereby enhancing selection on effort (47). They also had a significantly improved structure in the dorsolateral prefrontal cortex. The larger gray matter volume in the dorsolateral prefrontal cortex mediates the association between the level of aerobic adaptation and executive function in humans (116). The structural improvements in the brain regions can effectively promote executive function. A lower concentration of oxyhemoglobin in the dorsolateral prefrontal lobe during the recovery period was observed by fNIRS after moderate and high-intensity intermittent exercise. This phenomenon contributes to enhanced oxygen utilization and executive function in the brain. However, the executive function performance did not immediately improve after exercise but was enhanced after 10 min (117). This condition is due to the gradual metabolic recovery after exercise, and high levels of arousal during this time may contribute to cognition. In brain energy supply studies, the subjects’ improved executive function could be maintained for 40 min after a single high-intensity intermittent exercise session (118). Given that the brain’s uptake of glucose gradually decreases as the subject’s exercise intensity increases, lactate needs to be used to compensate for the increased energy required to maintain neuronal activity during high-intensity exercise. Blood lactate concentration is positively correlated with executive function in the brain (119). However, the link between blood lactate concentration and brain structure has not been fully discussed. On the psychological side, the chronic stress caused by the continuous involvement in online games in IA patients causes lower catecholamine levels in the resting state compared with healthy individuals. Psychological chronic stress induced by continuous engagement in online games causes lower levels of catecholamines in IA patients than in healthy individuals at rest. This condition also causes a sustained downregulation of receptors for adaptive responses, which results in cognitive impairment and impaired executive function (120). Although exercise can improve the catecholamine levels in humans, excessively high concentrations can instead inhibit the executive function (121). Therefore, interventions should be based on moderate-intensity aerobic exercise.

#### 6.2.3. Decision-making system

Decision-making is critical, and the quality of decisions determines the outcome, which depends on the level of rewards and execution systems. Patients with IA have impairments in the reward and executive systems that cause them to choose immediate rewards for small rewards for gratification over delayed rewards for large rewards when making decisions. Exercise appears to alter the efficiency and flexible regulation of neural circuits of cognitive control (122), thereby improving the decision-making system. Moreover, exercise can reduce or abstain from IA by improving the functional connections between networks of brain regions. Functional brain network connectivity was enhanced in subjects after the exercise intervention, and a significant correlation was observed between this functional brain network and the indicators of cognitive performance (123). However, the varying intensities of exercise have different effects on decision-making functions. The oxygenation function of the PFC was found to increase at low, medium, and high-intensity exercise, and the decision-making function also increased. However, this function decreases near or above the maximum intensity, with a consequent decrease in the decision-making function (124). Recent studies have shown that the insula and superior frontal gyrus cortical thickness are thinner in exercisers than in sedentary individuals. These regions are involved in autonomous brain control, stress response, and negative emotions (125), thus influencing decision-making functions. By contrast, exercise plays a positive role in voluntary activity, stress reduction, mood, and sensation (126, 127), thereby enhancing the insula and superior frontal gyrus cortices. The next pressing challenge is to explore the link between this positive effect and the thinner insula and superior frontal gyrus of exercisers.

## 7. Discussion

**FIGURE 2:**
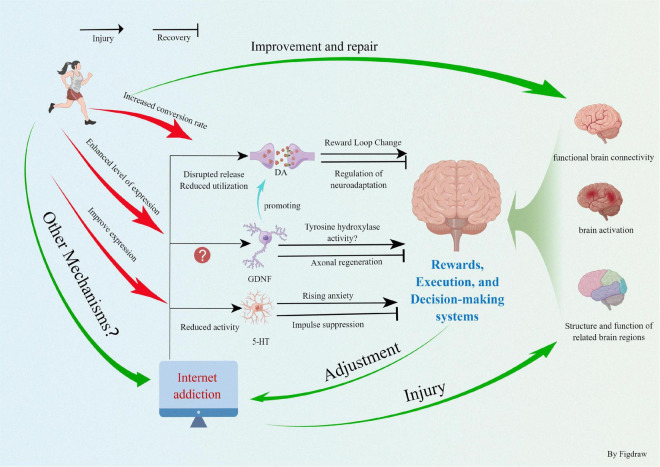
Exercise improves internet addiction and promotes physical health.

In summary, physical exercise can promote neurogenesis and angiogenesis in the brain by stimulating the specific cortical areas that induce the release of neurotransmitters and trophic factors. The behavioral and cognitive function in patients with IA must be improved. This work synthesizes that IA causes abnormal changes in the brain structure and function, thereby reducing the activity of the dopaminergic system and limiting oxygen utilization and functional connectivity of the brain. After we implemented the exercise intervention, the IA patients could increase the level of neurotrophic factors and neurotransmitters to a certain extent, stimulating the growth of neurons and cerebral blood vessels, which effectively improved the structure and function of the brain (Figure 2). The functioning of the three systems of reward, execution, and decision making was enhanced, the amount of time spent online was reduced, and the severity of IA was mitigated, thus improving the overall quality of life. Currently, the scientific field of exercise prescription interventions for IA and other addiction treatments is rapidly evolving. Exercise interventions do not have the side effects of pharmacological and other interventions, are easy to implement, and do not impose a financial burden on families and the society. However, several areas need to be addressed. (1) At the microscopic level, exercise directly or indirectly improves the impairment of brain function in IA patients by promoting the expression of neurotransmitters, such as DA and GDNF and their receptors. However, the complex structure of the neural network between IA and brain function impairment, whether more neurotransmitters are involved, and adaptive changes in the molecular structure during exercise intervention need to be explored and confirmed in depth. (2) To extensively explore the synergistic relationship between the reward circuit formed by IA and abnormal brain function. To observe the synergistic changes in exercise intervention and the adaptive changes in the psychological and physiological behaviors induced and reveal the changes in the reward system of IA patients restored by exercise and to improve abnormal brain function. (3) Exercise improves the executive and decision-making abilities of people with IA by promoting the brain structure, function (mainly the PFC system), and levels of various neurotransmitters. However, are there other associated brain regions or neural molecules? Is there a synergistic relationship between the corresponding brain regions? The deeper mechanisms behind the structural and functional patterns of the exercise facilitated brain remain to be explored. (4) In the future, we should organically integrate the macroscopic physiological behavioral manifestations with microscopic biomolecules. Together, they examine the fit pattern between neurotransmitters and brain function and behavior in IA patients and deeply explore the molecular adaptive changes brought about by motor behavior. In conclusion, with the increasing number of patients with IA, research on exercise interventions for IA should be continuously improved to provide a theoretical basis for future treatment and prevention.

## Author contributions

HC, GD, and KL contributed to the conception, designed the work, collected the information, and analyzed the data used in the systematic review. HC drafted the work. GD and KL substantively revised it. All authors read and approved the submitted version and agreed to be personally accountable for the authors’ contributions and to ensure the accuracy and integrity of the work.
